# Information on Meatox — Granulated Beef Fiber

**Published:** 1908-05

**Authors:** 


### Information on Meatox — Granulated Beef Fiber

H. ENDEMANN, New York, (*Der Hausdoktor,* April, 1908), remarks that our nourishing products, that is, the natural elements which are used as fool, are of two different kinds. We require heat or energy producing matter, such as starch, sugar and fat, and also matter which will build up our tissues and sustain our strength. For this latter purpose albumen and muscular fibers of meat are necessary, and these are transformed by the stomach and intestines into soluble substances which are assimilated and absorbed by the blood.

When the digestive tract is weakened, this process often goes on very slowly, particularly so on account of the common vice of swallowing food without proper mastication. For many years efforts have been made to counteract the effects of this fault, by offering to the public nourishing food in pulverised form. Starchy products when pulverised become flour, farina, etc., and fats become emulsions. For forty years this idea expressed itself in different methods applied to meat. My own investigations date back about that far, and I am satisfied that the digestibility of meat is considerably enhanced by its pulverisation. The older preparations, however, did not possess the very necessary qualities of retaining their strength and freshness.

Mr. Charles Marchand, of New York, has now found that the strength of such preparations is materially heightened when the extractive substances of the meat are first removed. These extractive substances are stimulant but they contain no nourishment, and the digestibility of the meat is not affected by their removal. This must be considered as an absolute advancement. The product obtained is known by the name of Meatox.

Meatox has from time to time been analysed by different chemists and it is absolutely free from preservatives. These analyses and physiologic experiments all show too that it contains a high percentage of digestible meat protein, that is, in round number about 80 per cent. Of fat it contains about 6 per cent. to 7 per cent., and of celery salt used as flavoring, less than 1 per cent., indigestible substances less than 1 per cent., with water and substances forming the balance.

That such a preparation has a very high value as food in cases of sickness and convalescence and in chronic malnutrition is obvi-of course. But it also fills a long felt want in that field where it becomes necessary to transport food products in a form of highest possible concentration and lightest weight, for instance in the provisioning of armies and navies, and of expeditions and traveling parties into wild countries. One pound of Meatox is equivalent in nourishing value to five pounds of lean, boneless beef, or the same quantity of "Canned Beef," which latter, when "preserved," often acts as a poison.

Meatox cannot make a bouillon, because the extractive substances are missing, but if the aroma is desired, it is easily obtained by adding a small quantity of meat extract, which will act as a stimulant, but it does not add any amount of nutritious substance whatever. I know of no meat preparation which possesses such a high percentage of digestible nutriment as does Meatox.

